# Usefulness of lower extremity pulse oximetry for detection of external iliac artery dissection during robot-assisted radical prostatectomy

**DOI:** 10.1186/s40981-025-00797-6

**Published:** 2025-06-11

**Authors:** Jun Honda, Akifumi Onagi, Ruriko Honda, Seiji Hoshi, Hidenori Akaihata, Satoki Inoue

**Affiliations:** 1 Department of Anesthesiology, Fukushima Medical University Hospital, 1 Hikarigaoka, Fukushima, Fukushima 960-1295 Japan; 2 Department of Urology, Fukushima Medical University Hospital, 1 Hikarigaoka, Fukushima, Fukushima 960-1295 Japan

**Keywords:** Robot-assisted radical prostatectomy, External iliac artery dissection, Pulse oximetry, Ultrasound, Complication

To the Editor

Pulse oximetry is a standard method of monitoring percutaneous oxygen saturation and pulse rate during surgery. In addition, it has been suggested to be a potential tool for detecting ischemia in the vessels of the lower extremities [1]. We here report a case in which pulse oximetry led to the detection of an intramural hematoma in the external iliac artery during robot-assisted radical prostatectomy (RARP).

A 71-year-old man (166 cm, 67 kg) was scheduled for RARP due to prostate cancer. He had hypertension as a comorbidity and was taking a fixed-dose combination of losartan and hydrochlorothiazide. After induction of general anesthesia, he was placed in the lithotomy position, and a pulse oximeter was attached to the right great toe. At the start of robotic surgery, the patient was placed in a 24-degree head-down position, and abdominal insufflation pressure was set at 10 cmH_2_O. The bilateral external iliac arteries were markedly tortuous. At 2 h and 30 min after right external iliac lymph node dissection, the right external iliac artery showed no discoloration, and pulse oximetry readings remained normal (Fig. 1). However, 40 min later, when the bladder and urethra were anastomosed and hemostasis was being checked, the perfusion index dropped from 0.78 to 0.15, and pulse oximetry measurement became unavailable; the attachment site was therefore changed to the earlobe. When the right external iliac artery was checked in the operative field, its color changed to a darker tone. Therefore, intraoperative ultrasonography was performed using robotic forceps, revealing a focal intramural hematoma and partial disruption of the lumen in the right external iliac artery (Fig. 2). If the blood flow obstruction persisted, we were planning to perform thrombectomy or angioplasty. However, follow-up intraoperative ultrasonography after 30 min of observation showed no hematoma enlargement. When the pulse oximeter was reattached to the right great toe, measurement became possible once again. The procedure was completed after verifying Doppler flow in the right dorsalis pedis artery. Three days postoperatively, the patient’s ankle brachial pressure index was within normal range at 1.18 on both sides, and it was indicating that there was no progression of hemodynamic disturbance.

**Fig. 1:**
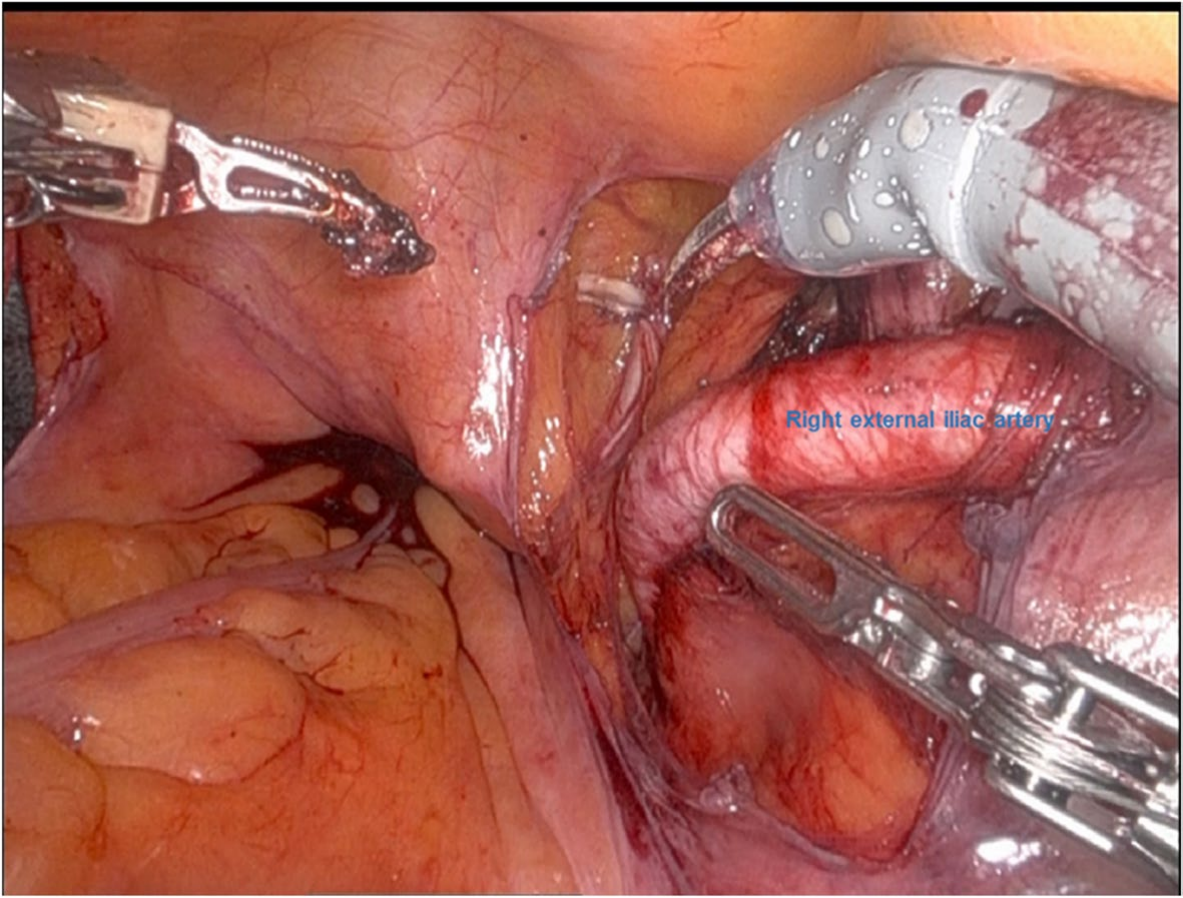
The right external iliac artery is normal in color

**Fig. 2:**
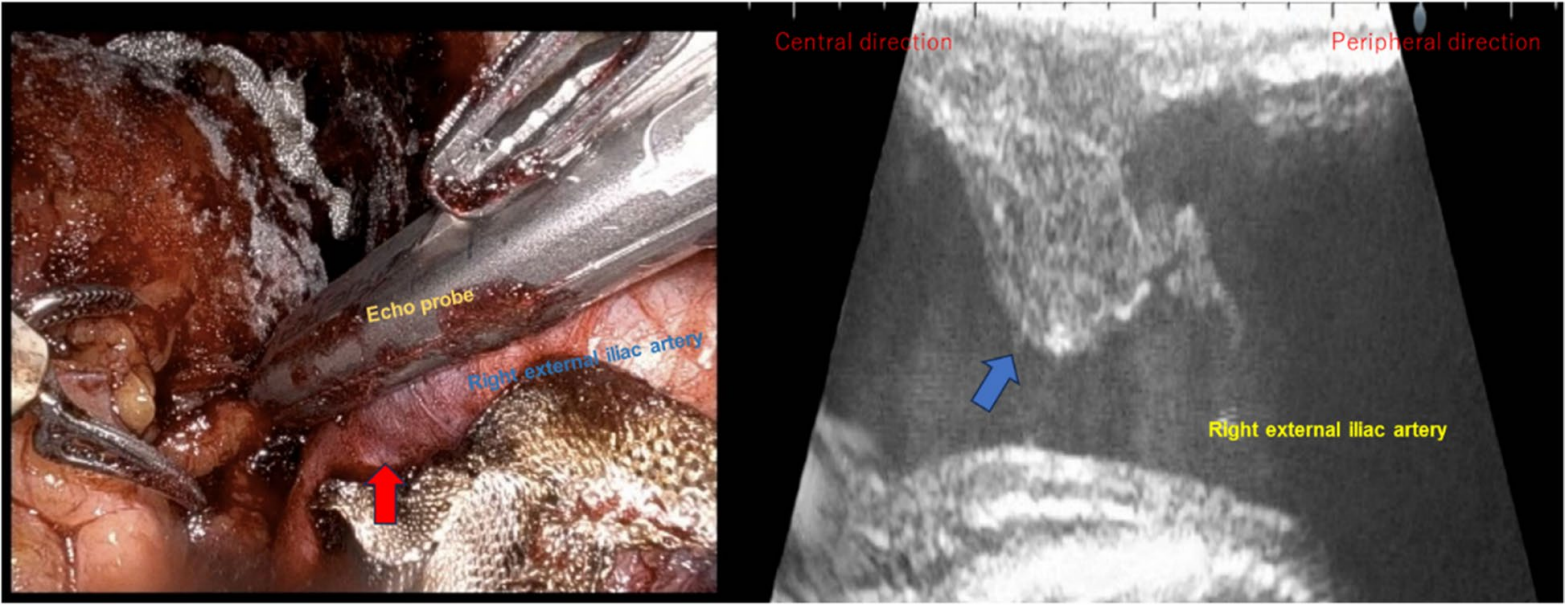
The right external iliac artery has a darker tone (red arrow). Ultrasound imaging shows an intramural hematoma with partial disruption on the ventral side of the external iliac artery lumen (blue arrow)

Since RARP involves abdominal insufflation in the lithotomy and Trendelenburg positions, decreased perfusion of the lower extremities, including complications of lower extremity compartment syndrome, has been reported [2]. At our institution, a pulse oximeter is usually placed on the lower extremity during RARP to monitor for circulatory disturbances due to lower extremity compartment syndrome. In the present case, the blood flow disturbance occurred in the lower extremity where the pulse oximeter was attached, allowing for its detection.

Although near infrared spectroscopy can also be used to monitor lower extremity blood flow [3], pulse oximetry remains the standard and cost-effective intraoperative monitoring tool, and should ideally be applied to both legs. While pulse oximetry is less accurate than Doppler ultrasound in detecting ischemic injury in the lower extremities, it has been reported to demonstrate accuracy comparable to the ankle-brachial index [1], and also provides the benefit of continuous monitoring. In addition, the perfusion index has been shown to serve as an indicator of progression to critical limb ischemia in patients with mild peripheral artery disease, which further supports the usefulness of pulse oximetry in detecting lower extremity hypoperfusion [4]. Although the cause of the intramural hematoma in this case is unknown, pelvic lymph node dissection may cause injury to the external and internal iliac vessels [5]. During RARP, the patient is placed in a steep head-down position, and intraoperative fluid administration is limited. Consequently, perfusion of the lower extremity may have been suppressed, making blood flow disturbances more easily detectable by pulse oximetry. The present case suggests the potential utility of lower extremity pulse oximetry in detecting vascular injury as well as lower extremity compartment syndrome during RARP.

## Data Availability

Not applicable.

## Abbreviation

RARP: Robot-assisted radical prostatectomy
